# Modulation of Sweet Taste by Umami Compounds via Sweet Taste Receptor Subunit hT1R2

**DOI:** 10.1371/journal.pone.0124030

**Published:** 2015-04-08

**Authors:** Jaewon Shim, Hee Jin Son, Yiseul Kim, Ki Hwa Kim, Jung Tae Kim, Hana Moon, Min Jung Kim, Takumi Misaka, Mee-Ra Rhyu

**Affiliations:** 1 Division of Creative Food Science for Health, Korea Food Research Institute, Bundang-gu, Sungnam-si, Gyeonggi-do, Republic of Korea; 2 Department of Applied Biological Chemistry, Graduate School of Agricultural and Life Sciences, The University of Tokyo, Bunkyo-ku, Tokyo, Japan; The University of Tokyo, JAPAN

## Abstract

Although the five basic taste qualities—sweet, sour, bitter, salty and umami—can be recognized by the respective gustatory system, interactions between these taste qualities are often experienced when food is consumed. Specifically, the umami taste has been investigated in terms of whether it enhances or reduces the other taste modalities. These studies, however, are based on individual perception and not on a molecular level. In this study we investigated umami-sweet taste interactions using umami compounds including monosodium glutamate (MSG), 5’-mononucleotides and glutamyl-dipeptides, glutamate-glutamate (Glu-Glu) and glutamate-aspartic acid (Glu-Asp), in human sweet taste receptor hT1R2/hT1R3-expressing cells. The sensitivity of sucrose to hT1R2/hT1R3 was significantly attenuated by MSG and umami active peptides but not by umami active nucleotides. Inhibition of sweet receptor activation by MSG and glutamyl peptides is obvious when sweet receptors are activated by sweeteners that target the extracellular domain (ECD) of T1R2, such as sucrose and acesulfame K, but not by cyclamate, which interact with the T1R3 transmembrane domain (TMD). Application of umami compounds with lactisole, inhibitory drugs that target T1R3, exerted a more severe inhibitory effect. The inhibition was also observed with F778A sweet receptor mutant, which have the defect in function of T1R3 TMD. These results suggest that umami peptides affect sweet taste receptors and this interaction prevents sweet receptor agonists from binding to the T1R2 ECD in an allosteric manner, not to the T1R3. This is the first report to define the interaction between umami and sweet taste receptors.

## Introduction

Most food products comprise of multiple mixtures of tastants. Animals integrate and unify the information regarding each separate taste and decide on their feeding behavior. Much research has focused on and described the interactions between taste modalities [1–4]. However, these studies are restricted to observations of phenotype and phenomenon and the detailed molecular and cellular mechanisms have not been fully investigated.

These interactions occur not only at the level of neuronal transduction but also at level of taste receptor [5, 6]. This crosstalk probably results from multiple mode of ligand binding to taste receptors. For example, a recent study revealed that binding of amiloride, a type of salt sensing reducer, to sweet receptors inhibited their responses [7].

Taste-taste interactions among the basic tastes have been investigated [1, 2]. Umami also interacts with the other tastes. Kemp and Beauchamp [8] concluded that at moderate/high concentrations of monosodium glutamate (MSG), sweet and bitter tastes were suppressed. Conversely, Woskow [9] reported that 5’-ribonucleotides which exhibit umami taste enhanced sweetness and saltiness at moderate concentrations, while sourness and bitterness were suppressed. Since these observations are based on behavioral indices, it remains to be elucidated whether the increase or decrease of sweetness caused by umami compounds occur at sweet taste receptor cells.

Sweet taste receptors in mammals are heterodimeric receptor complexes that comprise of T1R2 (taste type 1 receptor 2) and T1R3 (taste type 1 receptor 3) [10–12]. These receptors have a transmembrane domain (TMD) and a large extracellular domain (ECD), which is composed of a large extracellular venus flytrap domain (VFD) and a short cysteine-rich domain (CRD) [12,13]. Several reports show that the ECD is responsible for agonist recognition [14–17]. Aspartame and acesulfame K are recognized by the ECD of human T1R2 (hT1R2). In contrast, TMD of human T1R3 (hT1R3) is responsible for the recognition of cyclamate and for binding of lactisole which acts as a noncompetitive inhibitor [18–20].

In this study, we investigated the relationship between umami compounds—such as MSG and glutamyl dipeptides—and sweet receptors at the receptor level. We showed that umami compounds inhibited the response of sweet receptors in a manner dependent on the sweet receptor agonist. In addition, we provide the evidence that umami compound might inhibit agonist binding at T1R2 in allosteric manner.

## Materials and Methods

### Materials

Sucrose, acesulfame K, aspartame, cyclamate and MSG (L-glutamic acid monosodium salt hydrate) were purchased from Sigma-Aldrich (St. Louis, MO, USA). Glu-Glu, Glu-Asp were synthesized from Lugen Sci (Seoul, Republic of Korea). Cell culture media were obtained from Life Technologies, Inc. (Grand Island, NY, USA).

### Cell culture and transfection

Flp-In 293 cells stably expressing hT1R2, hT1R3 and αGustducin (wild-type) and hT1R2, hT1R3(F778A) and αGustducin (mutant) were prepared as described previously [7,21]. The hT1R2/hT1R3-expressing cells were maintained in Dulbecco’s modified Eagle’s medium (DMEM; Invitrogen, Carlsbad, CA, USA) containing 10% fetal bovine serum (FBS; Invitrogen) and 0.2% hygromycin B (Invitrogen). All cells were incubated at 37°C in a humidified atmosphere containing 5% CO_2_. Cultured hT1R2/hT1R3-expressing cells were seeded onto 96-well black-wall plates for 24 h prior to their use in experiments.

### Ca^2+^ imaging of the responses of hT1R2 and hT1R3-expressing cells

hT1R2/hT1R3 stably expressing cells were seeded onto 96-well black-wall imaging plates (BD Falcon Labware, Franklin Lakes, NJ, USA) for 24 h prior to their use in experiments. After 24 h, the cells were washed with assay buffer (130 mM NaCl, 10 mM glucose, 5 mM KCl, 2 mM CaCl_2_, 1.2 mM MgCl_2_ and 10 mM HEPES; pH 7.4) and loaded with the Ca^2+^ indicator dye Fluo-4 (5 μM; Invitrogen) in assay buffer for 30 min at 27°C. The cells were rinsed with assay buffer, incubated in 100 μL of assay buffer for > 10 min and then treated with ligand by adding 100 μL of the ligand solution. Fluo-4 was excited with the 486nm, and fluorescence was measured at wavelengths >515nm. [Ca^2+^]i was read into a computer-controlled filter changer (Lambda DG4; Sutter Instrument Co., San Rafael, CA, USA), an Andor Luca CCD camera (Andor Technology, Belfast, Northern Ireland) and an inverted fluorescence microscope (IX-71; Olympus, Tokyo, Japan). Images were recorded at 3-s intervals and were analyzed using the MetaMorph software (Molecular Devices, Sunnyvale, CA, USA).

### Measurement of cytosolic Ca^2+^ levels in hT1R2/hT1R3-expressing cells using a fluorescence plate reader

hT1R2/hT1R3 stably expressing cells were seeded onto 96-well black-wall CellBIND Surface plates (Corning Inc., Corning, NY, USA) for 24 h before the assay. The cells were loaded with 5 μM Calcium-4 (Molecular Probes, Eugene, OR, USA) in assay buffer for 45 min at 27°C. Subsequently, the cytosolic Ca^2+^ concentration was measured using a Flex Station III microplate reader (Molecular Devices). Sample solutions were loaded after a 17-s baseline scan and ligand-induced changes in fluorescence intensity (excitation, 485 nm; emission, 525 nm; cutoff, 515 nm) were monitored at 2.1-s intervals for 120 s. The response of each well is presented as the change in relative fluorescence units (ΔRFU), which was defined as the maximum minus the minimum fluorescence value. All experiments were performed at least three times.

## Results

### Umami compounds influenced the response of sweet receptor cells to sucrose

**Fig 1:**
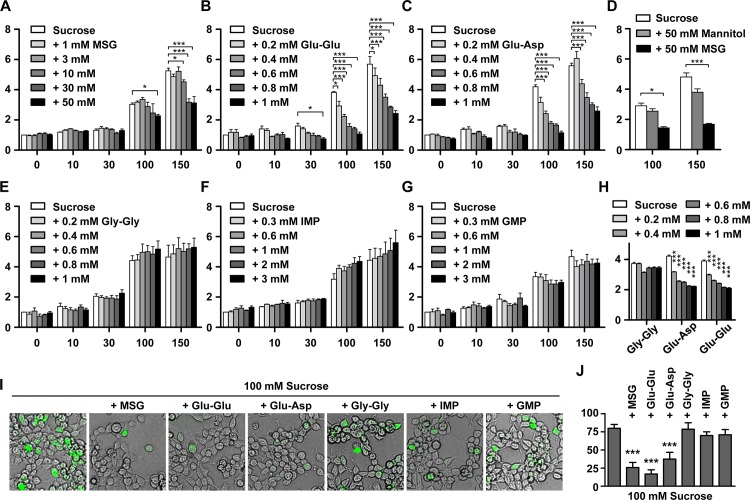
Inhibition of sucrose-induced calcium responses in hT1R2/hT1R3-expressing cells by MSG (A), Glu-Glu (B), Glu-Asp (C), Gly-Gly (E), IMP (F), and GMP (G). Glu-Glu and Glu-Asp are umami peptides and Gly-Gly is tasteless peptide. Inhibitory effect of umami compounds evaluated using the Flex system. Co-application of MSG or glutamyl dipeptides with sucrose inhibited the response of sweet receptor cells to sucrose. However, co-application of the 5’-ribonucleotides IMP and GMP with sucrose did not inhibit the response of sweet receptor cells to sucrose. **(D)** Osmolarity effects in hT1R2/hT1R3-expressing cells. **(H)** Sucrose response with dipeptides adjusted to pH 7.4. The value of y-axis means the ratio which the value with agonists and/or compounds normalized by the value without agonist. Asterisk *, **, *** stands for p<0.05, p<0.01, p<0.001, respectively. **(I)** Ca^2+^ cell images using fluo-4 dye is captured at 30 seconds later after co-applied sucrose and/or umami-compounds. Responded cell emitted the green fluorescence signals. Similar to the results of the Flex system, the response of sweet receptor cells to sucrose was attenuated by the co-application of Glu-Glu, Glu-Asp or MSG. **(J)** The ratio of responding cells to total cells.

To examine interaction between sweet and umami taste signaling at the receptor level, we evaluated the response of sweet receptor cells after treatment with various combinations of umami compounds and sucrose. The response of sweet receptor cells was monitored using the Flex system with the Ca4 dye. When MSG was co-applied with sucrose, the induced response of sweet receptor cells by sucrose was attenuated (Fig 1A). Other umami compounds; i.e., glutamate-glutamate (Glu-Glu) dipeptide, and glutamate-aspartate (Glu-Asp) dipeptide, also reduced significantly the response of sweet receptor cells (Fig 1B and 1C). To investigate whether all dipeptides might affect sweet receptor activity, we tested the sweet response with glycine-glycine (Gly-Gly) dipeptide which is well known as the taste-less dipeptides. Gly-Gly did not show inhibitory effect as induction of sweet receptor by sucrose (Fig 1E). Attenuated effect by MSG or umami dipeptides got clear as strong induction by treatment of high concentration of sucrose. Since treatment of sucrose in higher concentration than 150 mM had the effect on only Flp-In 293 cells without expressing sweet receptors, we did not perform the experiments with higher than 150 mM sucrose. The inhibitory effect of MSG and dipeptides showed dose-dependency (Fig 1A–1C). Because high osmolarity with MSG might cause inhibitory effect of sucrose response, we performed the control experiment using high concentration of Mannitol to mimic high osmolarity [22]. 50 mM of Mannitol causes the inhibited sucrose response. However, it seems to be ignorable because the inhibitory rate by Mannitol is much less than that by MSG (Fig 1D). In addition, low pH with acidic dipeptides might cause inhibited sucrose response. To remove the pH effect, we checked the response of sucrose with dipeptides revised to pH 7.4 using NaOH [23]. They are still highly effective in the inhibited sucrose response as much as unrevised dipeptides are (Fig 1H). Sweet receptor signaling finally increases cellular Ca^2+^ level via cAMP or IP_3_ production [24]. Although the change of cellular Ca^2+^ level was detected by Flex system, this phenomenon was more convinced by visualization of cellular Ca^2+^ level using imaging system with the Fluo-4 dye (Fig 1I and 1J). Visualized images using Fluo-4 dye also showed the inhibited sucrose response by MSG or dipeptides. These results were quantified by measuring the ratio of fluorescent cells by total cells in the Fig 1J. To investigate whether all umami compounds could inhibit sweet receptor activity, we tested the response of sweet receptor cells after treatment with 5’-ribonucleotide umami agonists such as IMP and GMP (Fig 1F and 1G). However, the 5’-ribonucleotide umami compounds did not reduce the response of sweet receptor cells to sucrose. Since 5’-ribonucleotide affects the T1R1 receptor, a component of the umami receptor complex, understandably the agonists had no effect on the T1R2/T1R3 sweet receptor complex.

### The inhibitory effect of umami compounds is dependent on the sweet receptor agonist

**Fig 2:**
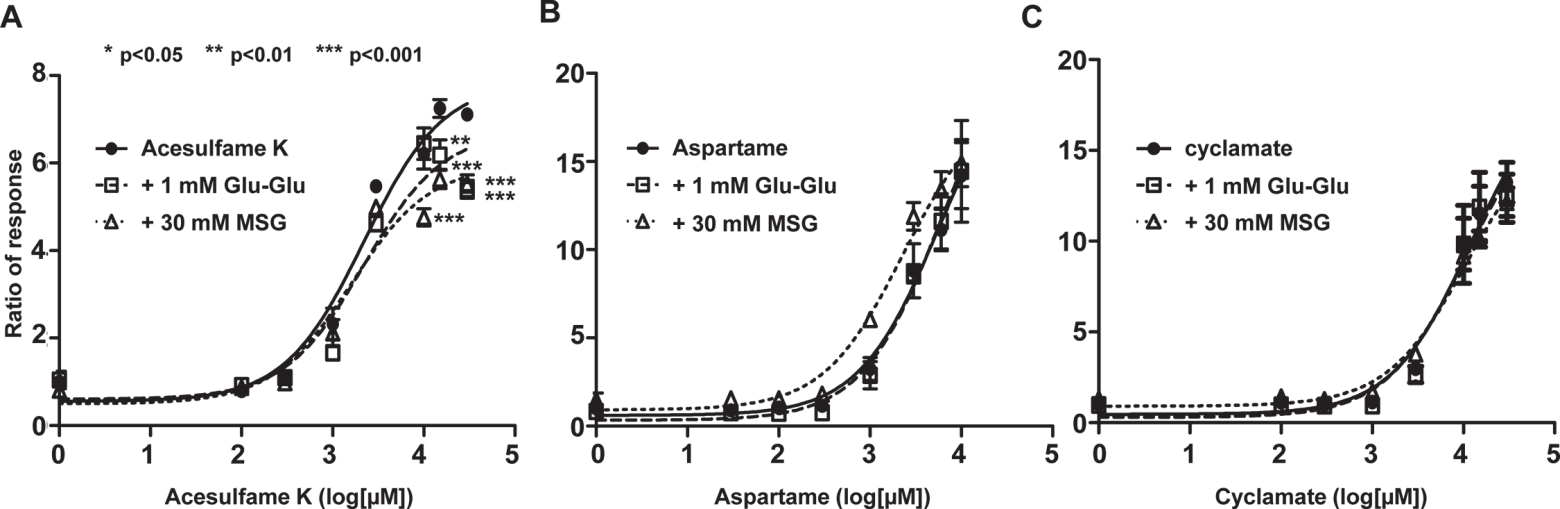
The inhibitory effect by umami compounds was dependent on the sweet receptor agonist. The responses of hT1R2/hT1R3-expressing cells were measured after co-application of umami compounds (30 mM MSG and 1 mM Glu-Glu) with other agonist; acesulfame K **(A)**, aspartame **(B)** or cyclamate **(C)**. Inhibitory effect of sweet receptor by MSG or umami dipeptide (Glu-Glu) was not shown at induction by aspartame or cyclamate. Asterisk *, **, *** stands for p<0.05, p<0.01, p<0.001, respectively.

To investigate whether MSG and umami-dipeptide inhibit agonist—receptor binding, we tested three other sweet receptor agonists: acesulfame K, aspartame and cyclamate. Acesulfame K and aspartame activate the sweet receptor in the ATD of T1R2, whereas cyclamate affects the TMD of T1R3. Induction of sweet receptor cells by acesulfame K was inhibited by Glu-Glu and MSG, similar to sucrose (Fig 2A). Compared with these agonists, treatment of Glu-Glu, MSG did not inhibit the cyclamate- or aspartame-induced response of sweet receptor cells (Fig 2B and 2C). Although aspartame affects the ATD of T1R2, a recent study suggested that aspartame uses different binding sites than acesulfame K [19]. These results suggest that MSG and umami dipeptides affect specific regions of the ATD of T1R2 to inhibit agonist binding.

### Umami compounds prevent sucrose binding to sweet receptors in an allosteric manner

**Fig 3:**
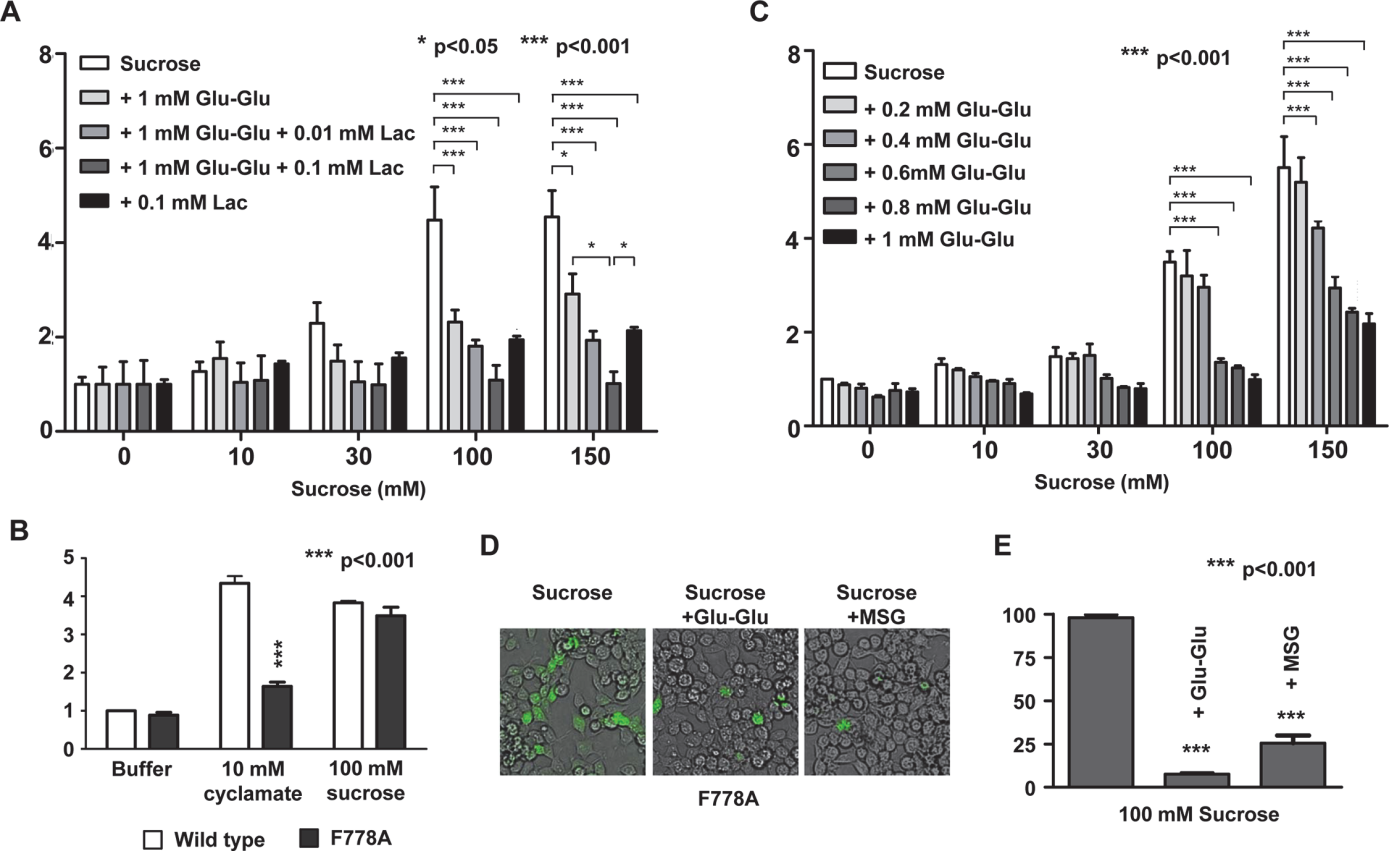
The target site of the umami peptide in sweet receptors was distinct from that of lactisole **(A)** The synergistically reduced response of sweet receptor cells upon application of sucrose. Co-application of 1 mM Glu-Glu with indicated concentration of lactisole (Lac) synergistically inhibited the sweet response. Asterisk *, **, *** stands for p<0.05, p<0.01, p<0.001, respectively. **(B)** The response of F778A mutant to cyclamate and sucrose. F778A means a point mutation at residue 778 of hT1R3 resulting in substitution of phenylalanine for alanine. **(C)** The response to the co-application of sucrose and Glu-Glu in cells expressing F778A mutant human sweet-taste receptors. **(D)** Imaging of co-application of 100 mM sucrose with 1 mM Glu-Glu or 50 mM MSG in cells with the F778A mutant receptor. **(E)** The ratio of responding cells to total cells.

Lactisole is the well-known antagonist by affecting the TMD of T1R3 [25]. To evaluate whether umami compounds works on the TMD of T1R3 for inhibition of sweet receptors, we performed a combination treatment of Glu-Glu and/or lactisole with sucrose. If umami compounds and lactisole share a common interacting surface in the TMD of T1R3, their inhibitory effect might be competitive. If umami compounds works on another site, the inhibitor effects would be additive or synergistic. Since no inhibitory effect of Glu-Glu on cyclamate (which also affects T1R3 TMD) was observed (Fig 2C), we hypothesized that the inhibitory mechanism of Glu-Glu might be different from that of lactisole. As expected, combination treatment of Glu-Glu and lactisole showed a reduced response to sucrose compared to that of each compound alone (Fig 3A). Additionally, we evaluated the inhibitory effect of the response of sweet receptor cells containing a F778A point mutation in T1R3. As a previous report [26], sweet receptor cells containing this point mutation showed defective cyclamate induction, whereas it did not show defective sucrose induction (Fig 3B). Combination treatment with Glu-Glu and sucrose in cells with this mutant receptor still showed the inhibitory effect of Glu-Glu (Fig 3C). These results indicate that the effect of Glu-Glu is not mediated *via* F778 on T1R3 TMD. Even though the results from Flex system were consistent and convincing, confirmation by the other system such as imaging system using the Fluo-4 dye would make our results stronger. This phenomenon was confirmed using an imaging system that could detect Ca^2+^ influx caused by receptor activity (Fig 3D). These results were quantified by measuring the ratio of fluorescent cells by total cells (Fig 3E).

## Discussion

Although much research has focused on taste-taste interactions, few studies have investigated the combination of sweet and umami tastants. Sako *et al*. [27] measured the response of the chorda tympani (CT) nerve of rodents to sucrose and MSG either alone or in combination with MSG, and reported that the combination of the sweeteners and MSG exerted synergistic effects. The enhancement was suggested to be due to colocalization of sweet and umami receptors in the same taste receptor cells (TRCs). Kemp and Beauchamp [8] reported changes in other tastes with the application of MSG based on behavioral testing. They showed that at moderate/high concentrations of MSG, sweet and bitter were suppressed; moreover, at a high concentration of MSG, the saltiness of NaCl was enhanced. However, these studies were based on the observation of behaviors and do not provide information regarding the underlying molecular mechanisms.

In this study, we investigated the relationship between umami compounds and the sweet receptor T1R2 and T1R3 complexes. Interestingly, MSG and glutamyl peptides inhibited the responses of sweet receptors. The reduced response of sweet receptor cells caused by umami compounds was dependent on the agonist type. Activation of sweet receptors by sucrose or acesulfame K was attenuated by umami compounds, whereas no such inhibition was detected when activated by cyclamate or aspartame. Ligands that are inhibited by umami compounds have a common activation site: the ECD of T1R2. Activation of sweet receptors by aspartame was not blocked, although aspartame induces the sweet receptor complex *via* the ECD of T1R2. This result can be explained based on the findings of a recent study [28]. Masuda *et al*. analyzed the binding modes between human sweet receptors and sweet compounds and defined the T1R2 residues used in agonist binding. In that report, sweet compounds were grouped according to the hT1R2 residues required for their recognition. Acesulfame K requires the R383, D142 and E382 residues of hT1R2 to activate sweet taste receptors, whereas Aspartame recognition requires the E302 and S144 residues of hT1R2. Because activation of sweet receptor with Acesulfame K was inhibited by umami compounds, but not with Aspartame, this allosteric hindrance of umami compounds might affect the R383 and D142 residues of the ECD of hT1R2.

Both of hT1R1 and hT1R2 belong to class C G protein-coupled receptors, thus sharing conserved structural features [29]. Each of these receptors possesses a large extracellular Venus flytrap domain (VFT) that is linked to a small extracellular cysteine-rich domain (CRD) and a seven-transmembrane domain (TMD). VFT consists of two lobes and the ligand binding site is located in a hinge region between the two lobes [19]. hT1R1 shows amino acid sequence homology with hT1R2 [30]. Based on well-known fact that MSG interact with VFT of hT1R1, this structural similarity supply the possibility that MSG might influence on hT1R2.

An alternative mechanistic hypothesis is that chemical interactions occur in the mixture before it comes into contact with the taste receptors. However, this is unlikely because the two structurally different types of agonist tested showed a common inhibitory effect.

In our study the underlying molecular mechanism could not be determined. Since we used only *in vitro* systems, examination of the interaction between umami compounds and sweet receptors might require different conditions. Additionally, the T1R2 residues to which umami peptides bind should be explored in further studies. Despite the limitations, our study is important because it is the first report to describe the relationship between sweet receptors and umami compounds at the molecular and receptor levels.

## References

[1] Temussi PA. Sweet, bitter and umami receptors: a complex relationship. Trends Biochem Sci. 2009;34: 296–302. 10.1016/j.tibs.2009.02.005 19443222

[2] Keast SJR, Breslin PAS. An overview of binary taste-taste interactions. Food Quality and Preference 2003;14: 111–124.

[3] Breslin PAS. Interactions among salty, sour and bitter compounds. Trends in Food Science & Technology 1996;7: 390–399.

[4] Tokita K, Boughter JD. Sweet-bitter and umami-bitter taste interactions in single parabrachial neurons in C57BL/6J mice. Journal of Neurophysiology 2012;108: 2179–2190. 10.1152/jn.00465.2012 22832571 PMC3545017

[5] Chu B, Chui V, Mann K, Gordon MD. Presynaptic Gain Control Drives Sweet and Bitter Taste Integration in *Drosophila* . Current Biology 2014;24: 1978–1984. 10.1016/j.cub.2014.07.020 25131672

[6] Kim MJ, Son HJ, Kim Y, Misaka T, Rhyu MR. Umami-bitter interactions: The suppression of bitterness by umami peptides via human bitter taste receptor. Biochemical and Biophysical Research Communications 2015;456: 586–590. 10.1016/j.bbrc.2014.11.114 25490385

[7] Imada T, Misaka T, Fujiwara S, Okada S, Fukuda Y, Abe K. Amiloride reduces the sweet taste intensity by inhibiting the human sweet taste receptor. Biochemical and Biophysical Research Communications 2010;397: 220–225. 10.1016/j.bbrc.2010.05.088 20493823

[8] Kemp SE, Beauchamp GK Flavor Modification by Sodium-Chloride and Monosodium Glutamate. Journal of Food Science 1994;59: 682–686.

[9] Woskow MH. Selectivity in flavor modification by 5'-ribonucleotides. Food Technology 1969;23: 32–37.

[10] Liman ER, Zhang YV, Montell C. Peripheral Coding of Taste. Neuron 2014;81: 984–1000. 10.1016/j.neuron.2014.02.022 24607224 PMC3994536

[11] Chandrashekar J, Hoon MA, Ryba NJP, Zuker CS. The receptors and cells for mammalian taste. Nature 2006;444: 288–294. 17108952 10.1038/nature05401

[12] Cui M, Jiang P, Maillet E, Max M, Margolskee RF, Osman R. The heterodimeric sweet taste receptor has multiple potential ligand binding sites. Curr Pharm Des. 2006;12: 4591–4600. 17168764 10.2174/138161206779010350

[13] Pin JP, Galvez T, Prezeau L. Evolution, structure, and activation mechanism of family 3/C G-protein-coupled receptors. Pharmacology & Therapeutics 2003;98: 325–354. 12782243 10.1016/s0163-7258(03)00038-x

[14] Assadi-Porter FM, Maillet EL, Radek JT, Quijada J, Markley JL, Max M. Key Amino Acid Residues Involved in Multi-Point Binding Interactions between Brazzein, a Sweet Protein, and the T1R2-T1R3 Human Sweet Receptor. Journal of Molecular Biology 2010;398: 584–599. 10.1016/j.jmb.2010.03.017 20302879 PMC2879441

[15] Liu B, Ha M, Meng XY, Kaur T, Khaleduzzaman M, Zhang Z, et al Molecular Mechanism of Species-Dependent Sweet Taste toward Artificial Sweeteners. Journal of Neuroscience 2011;31: 11070–11076. 10.1523/JNEUROSCI.0791-11.2011 21795555 PMC3149845

[16] Nie Y, Vigues S, Hobbs JR, Conn GL, Munger SD. Distinct contributions of T1R2 and T1R3 taste receptor subunits to the detection of sweet stimuli. Currernt Biology 2005;15: 1948–1952. 10.1016/j.cub.2005.09.037 16271873

[17] Li X. T1R receptors mediate mammalian sweet and umami taste. Am J Clin Nutr 2009;90: 733S–737S. 10.3945/ajcn.2009.27462G 19656838

[18] Jiang P, Cui M, Zhao B, Snyder LA, Benard LM, Osman R, et al Identification of the cyclamate interaction site within the transmembrane domain of the human sweet taste receptor subunit T1R3. Journal of Biological Chemistry 2005;280: 34296–34305. 16076846 10.1074/jbc.M505255200

[19] Xu H, Staszewski L, Tang H, Adler E, Zoller M, Li X. Different functional roles of T1R subunits in the heteromeric taste receptors. Proc Natl Acad Sci U S A. 2004;101: 14258–14263. 15353592 10.1073/pnas.0404384101 PMC521102

[20] Winnig M, Bufe B, Meyerhof W. Valine 738 and lysine 735 in the fifth transmembrane domain of rTas1r3 mediate insensitivity towards lactisole of the rat sweet taste receptor. BMC Neurosci. 2005;6: 22 15817126 10.1186/1471-2202-6-22 PMC1084349

[21] Fujiwara S, Imada T, Nakagita T, Okada S, Nammoku T, Abe K, et al Sweeteners interacting with the transmembrane domain of the human sweet-taste receptor induce sweet-taste synergisms in binary mixtures. Food Chemistry 2012;130: 561–568.

[22] Lyall V, Heck GL, DeSimone JA, Feldman GM. Effects of osmolarity on taste receptor cell size and function. American Journal of Physiology 1999;277: C800–13. 10516110 10.1152/ajpcell.1999.277.4.C800

[23] Sakurai T, Misaka T, Nagai T, Ishimaru Y, Matsuo S, Asakura T, et al pH-dependent inhibition of the human bitter taste receptor hTAS2R16 by a variety of acidic substances. Journal of Agricultural and Food Chemistry 2009;57: 2508–2514 10.1021/jf8040148 19231899

[24] Cygankiewicz AI, Maslowska A, Krajewska WM. Molecular Basis of Taste Sense: Involvement of GPCR Receptors. Critical Reviews in Food Science and Nutrition 2014;54: 771–780 10.1080/10408398.2011.606929 24345047

[25] Jiang P, Cui M, Zhao B, Liu Z, Snyder LA, Benard LM, et al Lactisole interacts with the transmembrane domains of human T1R3 to inhibit sweet taste. Journal of Biological Chemistry 2005;280: 15238–15246. 15668251 10.1074/jbc.M414287200

[26] Jiang P, Cui M, Zhao BH, Snyder LA, Benard LM, Osman R, et al Identification of the cyclamate interaction site within the transmembrane domain of the human sweet taste receptor subunit T1R3. Journal of Biological Chemistry 2005;280: 34296–34305. 16076846 10.1074/jbc.M505255200

[27] Sako N, Tokita K, Sugimura T, Yamamoto T. Synergistic responses of the chorda tympani to mixtures of umami and sweet substances in rats. Chemical Senses 2003;28: 261–266. 12714449 10.1093/chemse/28.3.261

[28] Masuda K, Koizumi A, Nakajima K, Tanaka T, Abe K, Misaka T, et al Characterization of the modes of binding between human sweet taste receptor and low-molecular-weight sweet compounds. PLoS One 2012;7: e35380 10.1371/journal.pone.0035380 22536376 PMC3335050

[29] Kniazeff J, Prezeau L, Rondard P, Pin JP, Goudet C. Dimers and beyond: The functional puzzles of class C GPCRs. Pharmacology & Therapeutics 2011;130: 9–25. 21256155 10.1016/j.pharmthera.2011.01.006

[30] Hoon MA, Adler E, Lindermeier J, Battey JF, Ryba NJ, Zuker CS. Putative mammalian taste receptors: a class of taste-specific GPCRs with distinct topographic selectivity. Cell 1999;96: 541–51 10052456 10.1016/s0092-8674(00)80658-3

